# Effects of Transcendental Meditation on mental health: a before-after study

**DOI:** 10.1186/1745-0179-4-25

**Published:** 2008-11-01

**Authors:** Masud Yunesian, Afshin Aslani, Javad Homayoun Vash, Abbas Bagheri Yazdi

**Affiliations:** 1 School of Public Health & Centre for Environmental Research, Medical Sciences, University of Tehran, Tehran, Iran; 2 School of Public Health, Medical Sciences, University of Tehran; Poursina Street, Keshavarz Boulevard, P.O. Box 14155-6446, Tehran, Iran; 3 Department of Mental Health, Ministry of Health and Medical Education, Tehran, Iran

## Abstract

**Background:**

Transcendental Meditation is a mental practice to put the body and mind into a state of relaxation and rest. The method was shown to reduce anxiety and stress in previous reports. This study investigates its potential benefits in enhancing mental health of an adult Muslim population.

**Methods:**

A before-after clinical trial was conducted to evaluate the effect of a 12-week meditation course on mental health of participants who were enrolled into the study by random sampling. 28-item General Health Questionnaire (GHQ) was administered on two occasions in conjunction with a background data sheet.

**Results:**

Mean age of participants was 32.4; they were 70% female and 55% married. GHQ scores improved significantly after the meditation course (p value: < 0.001). The difference was also significant in all subgroups of the population studied. In subclass analysis of the GHQ results, the before-after score improvement was significant only in the areas of somatisation (p value: < 0.001) and anxiety (p value: < 0.001).

**Conclusion:**

Transcendental Meditation may improve mental health of young adult population especially in the areas of somatisation and anxiety, and this effect seems to be independent of age, sex and marital status.

## Background

Meditation may be defined as the self-regulation of attention to suspend involvement in the habitual stream of thoughts. The goal of meditation is to reach a state of "thoughtless awareness," during which a person is passively aware of sensations at the present moment. Although there are a number of different techniques the elements of muscle and 'logic' relaxation, self-induced state and self-focused skill are considered essential [1]. The practice is believed to result in a state of physical and mental relaxation.

The form of meditation which is widely applied and studied in clinical medicine is Transcendental Meditation also known as TM. The technique which originated in the Vedic tradition in India uses a mantra (a word, sound, or phrase repeated silently) to prevent distracting thoughts from entering the mind. The intent of TM is to allow the mind to settle into a quieter state and the body into a state of deep rest. This is seen as ultimately leading to a state of relaxed alertness. The introduction of TM to Western world attracted the attention of psychotherapists and a relatively large number of research works are done to investigate the potential benefits of the method on mental health. The current literature emphasizes the role of the TM in reducing stress and anxiety [2,3]. However, little is known about the process by which meditation-based interventions may bring therapeutic benefits [4].

The current study evaluates the effects of TM on mental health of adults in Tehran, capital of Iran, with a predominantly Muslim population. According to a nationwide survey with the Persian version of GHQ-28 questionnaire, the level of mental distress in the country stands at 21%, with women being at greater risk than men (29% versus 15.8%), and depression and anxiety symptoms are more prevalent than somatisation and social dysfunction [5]. A similar survey which was conducted in the city of Tehran has reported 21.5% of the citizens to be at risk of mental disorders [6]. Considering the high prevalence of the mental distress in our society and the reported benefits of meditation in reducing anxiety and stress, this study was conducted to explore the possible effects of this technique on different areas of mental distress and its potentials for improving mental health of the society and widespread application in psychotherapy consultations in Iran.

## Methods

### Aim and design

The study was a before-after clinical trial to see whether a 12 week course of Transcendental Meditation has any significant effect on mental health of trainees or not. Four areas of mental health; anxiety, somatisation, depression and social dysfunction, were assessed. Similarly, the associations of any improvement with demographic characteristics of the sample were investigated. The study group were tested on two occasions; once just after the recruitment in the study and once upon completion of the course.

### Participants

The subjects were recruited from new members of the certified yoga clubs of Tehran with random sampling. These clubs hold famous reputable courses from more than 30 years ago and the courses are mentored by well-known instructors. Individual attendees in these courses have monthly follow up meetings with mentors and they discuss their personal experiences in TM and their problems are dealt with. Because of the excellent care, the clients generally complete the courses they have started in these clubs. The sample were 20 to 55 years old and had taken no more than three sessions at the clubs prior to enrolment in this study. Among the trainees those who satisfied these conditions were assigned numerical codes and 85 of them were selected by adding up the sampling interval to the first randomly selected figure – that is between one and the interval.

### Intervention

The participants attended a 12 week course from November 2005 until January 2006. There was an initial intensive training for first week consisting of: two introductory lectures (each 1.5 hours), a personal interview (15 – 20 minutes), a personal instruction session (1 – 1.5 hours) and daily program of group meetings which included three consecutive 30 minute practice sessions. In each practice session there was (1) concentration for one minute on respiration, (2) mantra meditation for 15 – 20 minutes: the mantras were assigned by mentors based on psychological traits of the attendees and they were different, (3) concentration for one minute, and (4) a rest for 10 minutes, in this order. After the first training week, they followed regular twice daily practice sessions at home in the morning and evening consisting of (1) concentration for one minute, (2) mantra meditation for 15 – 20 minutes, and (3) concentration for one minute for the rest of the 12 week study period. Follow up and maintenance meetings consisted of twice weekly group meetings (each 1.5 hours) during the study time. In addition, individual attendees were offered personal sessions for discussing their problems with TM mentors whenever they required. Participants received the same training on the meditation technique and followed similar practicing schedules at all clubs. Compliance with TM program was assessed by class attendance. Trainees did not receive any other medication or therapy such as Yoga Asana or Ayuravedic treatment alongside their TM course.

### Measures

The 28 item General Health Questionnaire (GHQ-28) which is validated in Iranian population was used as a self-administered tool for assessment of general mental health and mental distress in the four areas of depression, anxiety, somatisation and social dysfunction [7]. GHQ-28 asks about the presence of a range of symptoms during the last one month in the four relevant areas. It has 28 items and the answer to each item has four scales of 0: 'not at all', 1: 'same as usual', 2: 'more than usual' and 3: 'much more than usual'. Respondents' score is calculated by adding up the ratings of individual items and overall scores of 22 or higher are considered positive for mental distress. At this cutting score, the Persian version of GHQ-28 in Iranian population has sensitivity and specificity of 84.7%, 93.8% respectively [7]. The questionnaire was handed out in conjunction with a personal data sheet asking about age, sex and marital status. Although the questionnaire was filled anonymously the participants were given the chance of having their results by writing down a personal five digit code on the paper.

### Statistical methods

The mean GHQ scores of the participants in the two occasions were compared by Wilcoxon rank score test. The associations of scores with independent variables were tested by Chi square test. All calculations were performed by SPSS^®^.

## Results

A total of 80 members of the clubs completed the course and questionnaires (5 dropouts, response rate: 94%). The participants were a young group between 20 and 55 years old (Median: 31, Mean: 32.4), 70% female and 55% married. Compliance with the TM program based on class attendance was 98% for the group who completed the course. Overall, 38.8% of the sample had mental distress at the time of enrolment as assessed by GHQ-28. This figure decreased to 26.3% in the end of the 12 week course. There were not significant differences for mental distress prevalence in subgroups of age (Chi-square p value: 0.421), sex (p value: 0.249) and marital status (p value: 0.982) in our sample (see Table 1).

**Table 1:** Frequency of mental distress in subgroups of the sample at the start of the TM course and GHQ subclass scores before and after the course and levels of significance for improvement of test scores

	Subgroup n (%)	GHQ ≥ 22 n (%)	Mean GHQ subclass scores (before – after)				GHQ-28 score
				
			*Somatisation*	*Anxiety*	*Social Dysfunction*	*Depression*	
*Total*	80	31 (39%)	(6.2 – 4.8) p* < 0.001	(6.6 – 4.7) p < 0.001	(9.2 – 9.4) p = 0.979	(3.0 – 2.0) p = 0.152	(25.1 – 21.3) p < 0.001
*Age Group*							
20 – 29	35 (44%)	13 (37%)	(6.1 – 4.9) p < 0.001	(6.8 – 5.5) p < 0.001	(8.9 – 8.9) p = 0.979	(3.9 – 3.0) p = 0.152	(25.7 – 22.3) p < 0.001
30 – 39	25 (31%)	10 (40%)	(7.5 – 5.5) p = 0.019	(7.7 – 4.7) p = 0.006	(8.5 – 10.1) p = 0.352	(3.5 – 1.9) p = 0.106	(27.2 – 22.4) p = 0.028
≥ 40	20 (25%)	8 (40%)	(6.0 – 4.6) p = 0.023	(6.6 – 3.8) p = 0.036	(10.4 – 9.8) p = 0.292	(1.8 – 1.5) p = 0.733	(24.9 – 19.7) p = 0.022
*Gender*							
Male	24 (30%)	7 (29%)	(5.6 – 3.8) p = 0.005	(5.3 – 3.7) p = 0.029	(8.5 – 9.2) p = 0.667	(2.4 – 1.5) p = 0.094	(21.9 – 18.3) p = 0.01
Female	56 (70%)	24 (43%)	(6.5 – 5.3) p = 0.018	(7.2 – 5.1) p < 0.001	(9.5 – 9.6) p = 0.839	(3.3 – 2.7) p = 0.517	(26.4 – 22.7) p = 0.003
*Marital Status*							
Married	44 (55%)	17 (39%)	(5.8 – 4.7) p = 0.025	(6.1 – 4.1) p = 0.01	(9.3 – 9.8) p = 0.793	(2.7 – 2.0) p = 0.253	(23.8 – 20.6) p = 0.024
Unmarried	36 (45%)	14 (39%)	(6.8 – 5.1) p = 0.006	(7.3 – 5.5) p = 0.003	(9.1 – 9.0) p = 0.742	(3.5 – 2.7) p = 0.350	(26.6 – 22.3) p < 0.001

* All p values are for Wilcoxon rank score test

After the TM course, mean GHQ scores of the sample improved in the areas of somatisation and anxiety but there was no significant change on depression and social dysfunction scores. The overall score of the GHQ-28 test was also improved significantly from 25.1 before the course to 21.3 after the course (p < 0.001). That is, the mean score moved from a figure above the cut-off level of 22 for mental distress to below this level. This is especially important from clinical point of view because this cut-off level defines those who most probably will have clinically discernable mental health problems. As shown in Table 1, the findings were similar across the subgroups of the sample and age, sex and marital status does not seem to determine the effects of TM on meditators.

## Discussion

This study revealed that in a young adult population Transcendental Meditation practices result in improved mental health which was most remarkable in the areas of anxiety and somatisation and this improvement seems to be independent of age, sex and marital status. The beneficial effect of TM on these two areas is not just statistically significant but also clinically plausible as a short course like the one used in this study is predicted to affect anxiety and somatisation by the well-defined calming and relaxing properties of TM. There are a number of reports with similar findings for TM and other meditation techniques. Improved mental health is shown to result from yoga practices in community based settings [8] and from TM among selected adult populations in previous studies [9,10]. TM practices is reported to reduce anxiety when Spielberger State-Trait Anxiety Inventory is applied [9]. There are also studies that focus on the effect of TM on mental distress demonstrating the effect of TM in experimental settings at reducing the levels of mental distress in subjects [11,12].

In a recent Cochrane review with only two studies included, the authors conclude that TM is comparable with other relaxation therapies for anxiety disorders [3]. However, there are two other systematic reviews with larger numbers of studies included that suggest TM to be markedly more effective than other meditation and relaxation therapies [2,13]. It may be argued that the superiority of the method in comparison to other mind-body techniques stems from the more restful state that the body enters in during TM as evidenced by EEG changes and peculiar physiological findings [9,14].

There are special challenges for research on meditation [15]. Similar implementation of meditation across the study group is one of the difficulties encountered. The current study satisfied this condition and ensured that the same method of TM with similar schedule was practiced in all clubs over the course of the study. On the other hand, there are still important limitations for this research that deserve being mentioned. The participants of this study were new members of the yoga clubs who may have had joined the club to improve their poor mental health or relieve their anxiety states. This expected benefit among the attendees and the fact that those who join such clubs in Iranian culture belong to higher socioeconomic groups of the society brings about reservations to the notion that TM may be used as a community based approach to improve the mental health of the society at large. Studies on random samples from the general population are needed to define the method's ultimate role in medical practice.

The validity of the GHQ itself as a screening tool is especially limited in patients with more chronic symptoms and those with more frequent social and interpersonal problems [16]. Despite these intrinsic limitations of the GHQ it remains a universally used and standard tool for situational analysis and before-after comparisons. More specifically designed questionnaires like SCL90-R may provide more detailed descriptions than the findings in this trial and we suggest them for future studies.

There are also limitations to the before-after design. Regression to the mean is often ignored in this design and this is particularly the case when subjects with extreme measurements are enrolled. Secular trends may also affect the findings during the course of the study [17]. Because of the absence of control group the inference that 'the improvement in GHQ scores was the result of the TM course' can not be made with certainty. In other words, any cause out of our control and not related to our intervention may well be in place. More rigorous designs should be used to establish a cause-effect relationship and a randomised controlled trial in which the control group receives some other form of mind-body training may suit the purpose.

It is difficult to evaluate whether the effects of TM on mental health maintains over time or not. This is partly because the meditators after the program will probably be different in terms of their adherence to TM. That is, some may continue with the previous schedule, some may develop their own time table and others might give off or embark on other methods. It may be possible to find an answer to this question with a large cohort of meditators who are meticulously followed for a reasonably long period of time.

## Conclusion

Meditation improves mental health of volunteer yoga club members and the effects are mostly on relieving somatisation and anxiety. The favourable outcomes of meditation appear to be independent of age, sex and marital status.

More researches with rigorous designs in long-term follow-up periods are needed to address the questions about the role of the Transcendental Meditation in clinical practice.

## Competing interests

The authors declare that they have no competing interests.

## Authors' contributions

AA suggested the conception, all authors (MY, AA, JHV, ABY) participated in design of the study, AA supervised data gathering; all analysed the data and interpreted the results. JHV drafted the manuscript and it was critically revised by all authors and they have given final approval of the version to be published.
